# Predicting sensitivity of recently harvested tomatoes and tomato sepals to future fungal infections

**DOI:** 10.1038/s41598-021-02302-2

**Published:** 2021-11-30

**Authors:** Sanja Brdar, Marko Panić, Esther Hogeveen-van Echtelt, Manon Mensink, Željana Grbović, Ernst Woltering, Aneesh Chauhan

**Affiliations:** 1 BioSense Institute, University of Novi Sad, 21000 Novi Sad, Serbia; 2 Wageningen University and Research, 6708 PB Wageningen, The Netherlands

**Keywords:** Information technology, Plant stress responses, Imaging and sensing, Near-infrared spectroscopy

## Abstract

Tomato is an important commercial product which is perishable by nature and highly susceptible to fungal incidence once it is harvested. Not all tomatoes are equally vulnerable to pathogenic fungi, and an early detection of the vulnerable ones can help in taking timely preventive actions, ranging from isolating tomato batches to adjusting storage conditions, but also in making right business decisions like dynamic pricing based on quality or better shelf life estimate. More importantly, early detection of vulnerable produce can help in taking timely actions to minimize potential post-harvest losses. This paper investigates Near-infrared (NIR) hyperspectral imaging (1000–1700 nm) and machine learning to build models to automatically predict the susceptibility of sepals of recently harvested tomatoes to future fungal infections. Hyperspectral images of newly harvested tomatoes (cultivar Brioso) from 5 different growers were acquired before the onset of any visible fungal infection. After imaging, the tomatoes were placed under controlled conditions suited for fungal germination and growth for a 4-day period, and then imaged using normal color cameras. All sepals in the color images were ranked for fungal severity using crowdsourcing, and the final severity of each sepal was fused using principal component analysis. A novel hyperspectral data processing pipeline is presented which was used to automatically segment the tomato sepals from spectral images with multiple tomatoes connected via a truss. The key modelling question addressed in this research is whether there is a correlation between the hyperspectral data captured at harvest and the fungal infection observed 4 days later. Using 10-fold and group k-fold cross-validation, XG-Boost and Random Forest based regression models were trained on the features derived from the hyperspectral data corresponding to each sepal in the training set and tested on hold out test set. The best model found a Pearson correlation of 0.837, showing that there is strong linear correlation between the NIR spectra and the future fungal severity of the sepal. The sepal specific predictions were aggregated to predict the susceptibility of individual tomatoes, and a correlation of 0.92 was found. Besides modelling, focus is also on model interpretation, particularly to understand which spectral features are most relevant to model prediction. Two approaches to model interpretation were explored, feature importance and SHAP (SHapley Additive exPlanations), resulting in similar conclusions that the NIR range between 1390–1420 nm contributes most to the model’s final decision.

## Introduction

Tomato is a popular and commercially important horticultural produce worldwide^1^. Quality of tomato depends on growing conditions and chain conditions like humidity and temperature, as well as crop handling during harvest and post-harvest processes (transport, packaging, storage, processing etc.)^2^. Like many other perishable fruits and vegetables, it is highly prone to post-harvest losses, reaching up to 30% in some developing countries^3^. Early detection of disease has the potential to prevent losses because early actions can be taken to limit bigger damages (see e.g.^4^).

Tomato is known to be highly susceptible to pathogenic fungi, such as *Penicillium*, *Aspergillus* and *Mucor*, which tend to attack crops with high moisture and nutrient content^5–8^. The weakening and damage to tomato tissue can be caused by specific environmental conditions (humidity and temperature) as well as due to poor product handling. This creates a potential entrance for fungal spores which, given appropriate germination conditions, may infect the stem, calyx, sepals, or tomato skin.

Often the tomato calyx (a collection of sepals) is the first part of the tomato where an infection becomes visible^9^. In European supermarkets fresh tomatoes are nowadays sold with the calyx attached. The presence of fungus on the green parts (the crown) of the tomatoes is seen as undesirable, even though the tomato fruit itself is not infected. At the tomato collection and packing centers, quality inspectors are capable of observing necrosis on the calyces and know that these have a higher risk for developing fungal infections. However, calyces which look healthy after harvest and pass the quality inspection can still develop fungal infections. It is therefore hypothesized that there is a correlation between the physiology of the calyx (prior to fungal infection) and their susceptibility to eventual fungal infection (Janse and Boerrigter^10^ and this study). The physiology of the calyx is likely to be influenced by various growing conditions like radiation during fruit set and fruit growth, relative humidity during cultivation, crop management, plant load (fruits/m2), and nutritional level of the crop^10^. Furthermore, post-harvest conditions also influence the physiology of the calyx. Development of a non-destructive method to assess the physiology of the calyx in relation to the susceptibility to fungal infection would provide additional support to quality inspectors. This work focuses on predicting susceptibility of individual tomato sepals to fungal incidence.

Various studies have applied machine learning and computer vision methods for detection and classification of diseases in tomatoes^11^. These methods have mainly been investigated to detect and identify several types of visible symptoms, such as *Bacterial Spot*^12^ or visible damage (caused by *early or late blight, septoria leaf spot*)^11^, usually based on color images^4,13–15^. Prince et al.^16^ combine depth, temperature and color information to train a Support Vector Machine (SVM) classifier to detect powdery mildew in tomato leaves. Mokhtar et al.^14^ also used SVM to classify tomato yellow leaf curl virus. Zhao et al.^17^ utilize Adaboost classifier with Haar features to identify tomatoes in unstructured plant growth environment. Yamamoto et al.^18^ explore regression trees, based on simple color features, to detect tomatoes of different maturity level during plant growth. Singh et al.^19^ provide a taxonomy of classical machine learning methods suitable for detecting biotic and abiotic stress traits for plant phenotyping. The techniques applied in these studies primarily follow traditional *image processing pipeline* which constitutes manual feature extraction/engineering and training classifiers on these features.

Recent research tends to focus on applied deep learning^20^, and in particular deep convolutional neural networks (CNNs). A key advantage of CNNs is that the features are no longer hand-crafted but learned directly from data. CNNs have been shown to outperform conventional classification methods in several studies. Atabay^21^ trained a CNN using deep residual learning approach on a subset of PlantVillage dataset^22^, achieving state of the art performance in classifying tomato plant leaf images based on the visible effects of diseases. Already mentioned work of Brahimi et al.^11^ used an image-dataset of 14828 tomato leaves to train a CNN to identify nine diseases, and reported an accuracy of 99.18%. Belal et al.^23^ achieved similar performance using a CNN on a public dataset of 9000 images to identify 5 types of diseases on tomato leaves.

The approaches discussed above focus on color images. However, color images are not always the most suitable data, limiting the discriminatory capability of a classification method. It has been demonstrated previously that Hyperspectral Imaging (HSI) can be used for non-destructive and non-invasive monitoring and measurement of stress, infections and diseases in several types of crops and food products—e.g. for monitoring *Fusarium* infection and mycotoxin presence in wheat kernels and flour^24^, detection of insect-damaged wheat kernels^25^, for measuring fungal infection severity in maize kernels^26^, wheat ears^27^, sugar beet^28,29^, potato virus Y damage in^30^, draught stress in maize^31^ and fruit quality inspection^32^ and other food applications^33^. Mahlein et al.^29^ study spectral reflectance and SAM (Spectral Angle Mapping) method for classifying different (symptoms of) sugar-beet diseases. Xie et al.^34^ demonstrate that spectral reflectance and texture features can be used for the detection of fungal diseases that harm tomato leaves. Zhang et al.^35^ observed 4 stages of severity of fungal disease on tomatoes, and reported that spectral images in Near-infrared (NIR) spectra (700–1300 nm) are better for disease detection than visible images (350–700 nm). A more challenging task of presymptomatic detection was tackled in recent studies on tobacco^36^ and tomato^37^ plant disease. Also see^38^ for a review of recent works exploring close-range HSI for plant disease detection.

Conditions causing stress to the calyx, lowering defense responses in the cells, or causing cell death are likely the reason for increased susceptibility to fungus. HSI, especially in the Near Infra-Red (NIR) range, has been shown to be sensitive to certain types of cell damage, such as bruises^39,40^, but has not yet been demonstrated for cell damage on sepal tips and for early detection of weak sepals. This is the focus of current work. HSI is investigated for predicting the susceptibility to fungal infection of a sepal right after the tomato is harvested, but prior to the visible onset of the infection.

For this purpose, an experimental procedure was designed wherein hyperspectral images were acquired from several batches of tomatoes prior to any visible evidence of fungal infection, potentially capturing any symptoms of sepal weakening/cell damage or early infection. The tomatoes were then introduced to conditions stimulating fungal germination and growth for multiple days. On the final day of the experiment, tomatoes are imaged (color images) for gathering visual evidence of fungal severity. The purpose behind this procedure is to identify if there is a correlation between hyperspectral data (captured before any observable infection) with the observable infection on the final day of the experiment.

In this study two machine learning approaches (Random Forest and XGBoost regression) were used to find a correlation between the spectral information from the first day of the experiment and the fungal severity on the last day. Reported results demonstrate that the predicted fungal severity correlates well with the ground truth estimates and a high proportion of the variance is explained. Instead of looking at these models as blackboxes, the focus was also on model interpretation. In this work, model interpretation is used to identify the most relevant spectral wavelengths contributing to fungal severity prediction.

The rest of the paper is organized as follows: next section describes the materials and methods, with a focus on data collection, experimental setup, and creation of the hyperspectral and color image datasets; this is followed by the section on the novel hyperspectral data processing pipeline for automatically extracting data corresponding to individual sepals; results of machine learning based models are reported in “Results” section, along with the model explanations; “Discussion” section discusses the results from chemometric and physiological perspective; and “Conclusion” section concludes the article and proposes ideas for future research.

## Materials and methods

### Tomatoes

For the purposes of this research, 6 batches of cocktail tomatoes, cultivar Brioso RZ, were obtained from 5 commercial growers based in The Netherlands and Belgium. The tomatoes were provided and delivered by the growers to Wageningen Food and Biobased Research (WFBR) institute at Wageningen University and Research. The representative of the growers and WFBR form part of the consortia of the Public Private Partnership project Humistatus, funded through Foundation TKI, Dutch Horticulture and Starting Materials. The tomatoes were provided by the growers for the research reported in this article, which was agreed upon beforehand with the representative of the growers and the rest of the Humistatus consortia. Furthermore, the harvest was conducted by the growers themselves, which complies with the relevant institutional, national, and international guidelines and legislation.

All tomatoes were harvested on 12-Dec-2017, collected and transported by the growers at room temperature on 13-Dec-2017 to Wageningen and stored overnight a 15°C until the next day. Capital letters from A to F are used as markers for the batches to distinguish growers or growing conditions. Batches A-E came from different growers, all grown in greenhouses (without supplementary lighting) where some of the last tomatoes were harvested (the crops were already at the end of their lives). Batches E and F came from the same grower, but different greenhouses. Batch F came from a crop which had just started producing (using supplementary lighting). The next day, tomatoes from each batch containing 5 or 6 tomatoes are pruned into six smaller trusses where each truss contains between three and four tomatoes. Table 1 summarizes this data.

**Table 1:** A summary of the 6 batches of tomatoes used in the experiments.

Batch labels	Tomatoes/sepals	Crop description
A	23/109	No supplemental light, end of crop harvest
B	24/119	No supplemental light, end of crop harvest
C	24/115	No supplemental light, end of crop harvest
D	24/118	No supplemental light, end of crop harvest
E	24/116	No supplemental light, end of crop harvest
F	24/111	Supplemental light, new crop (first) harvest

### Hyperspectral dataset

**Figure 1:**
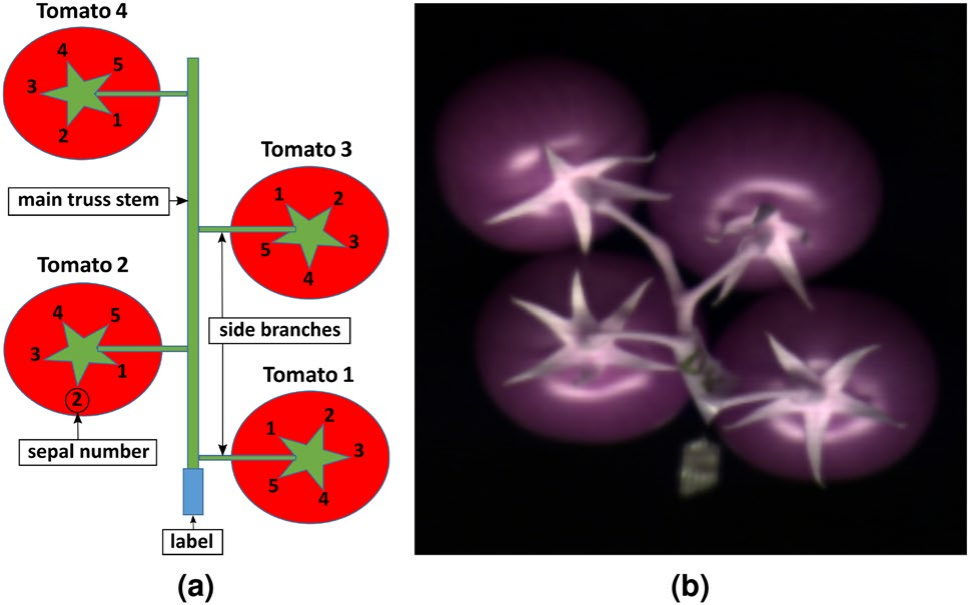
(**a**) The orientation of tomatoes during the hyperspectral imaging. (**b**) A pseudo color image of one truss obtained by combining three spectral bands.

The first part of the experiment involved hyperspectral data acquisition prior to any visible evidence of fungus on the tomatoes. Therefore, hyperspectral images were captured on the first day of the experiment (14-Dec-2017). Imaging was done using SPECIM FX17 camera, Specim, Finland. This instrument captures spectral responses in a range from 900–1700 nm with a resolution step of 3.46 nm, resulting in 224 bands/image. For the illumination source, two halogen lamps were used. Each truss was placed under the camera where the tip of the truss is facing towards the bottom and is marked using a blue tape (see Fig. 1 for an example).

### Treatment conditions and the color image dataset

**Figure 2:**
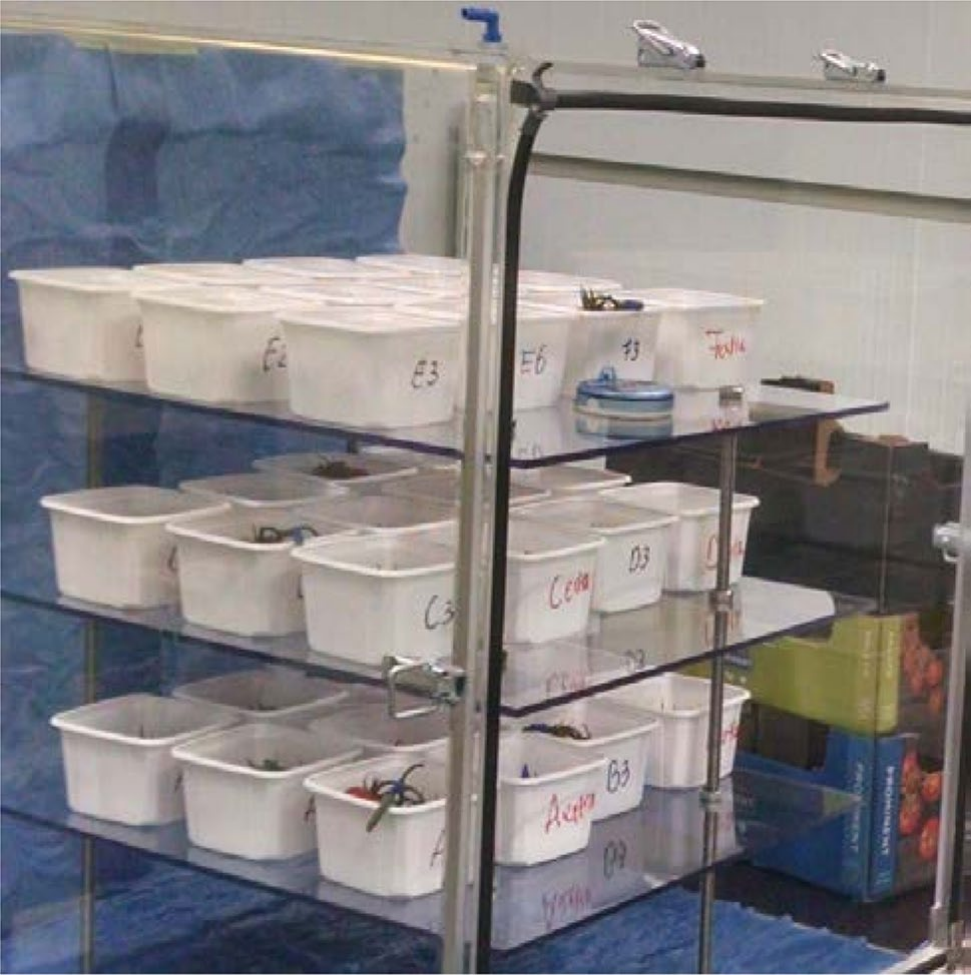
A controlled environment which is ideal for fungal germination.

In the second part of the experiment, right after hyperspectral data capture, tomatoes were placed in a controlled environment with a relative humidity of 100% (see Fig. 2). These conditions are ideal for fungal germination. The tomatoes were taken out of the controlled settings on 18-Dec-2017, and it was observed that almost all sepals had been infected to some degree (except Batch F).

To objectively capture the degree of infection, a reference data set was created with the RGB images of the trusses. This was done using the Smart Color Inspector^41^, SCI, which is a controlled and closed environment with calibrated light and camera settings to record true color of objects placed inside it. SCI is designed by WFBR and built by IPSS Engineering (both based in Wageningen, The Netherlands). It is mounted with LED arrays (4038 K) on five sides and is equipped with an RGB camera (MAKO G-192C POE, Allied Vision Technologies GmbH, Stadtroda, D) that takes images from above. For each measurement series, the system is calibrated with a white background (Forex$\circledR$ PVC Plate White 6 mm) and a 24-plane color chart (Color checker classic, X-rite Europe GmbH, Regensdorf, S).

**Figure 3:**
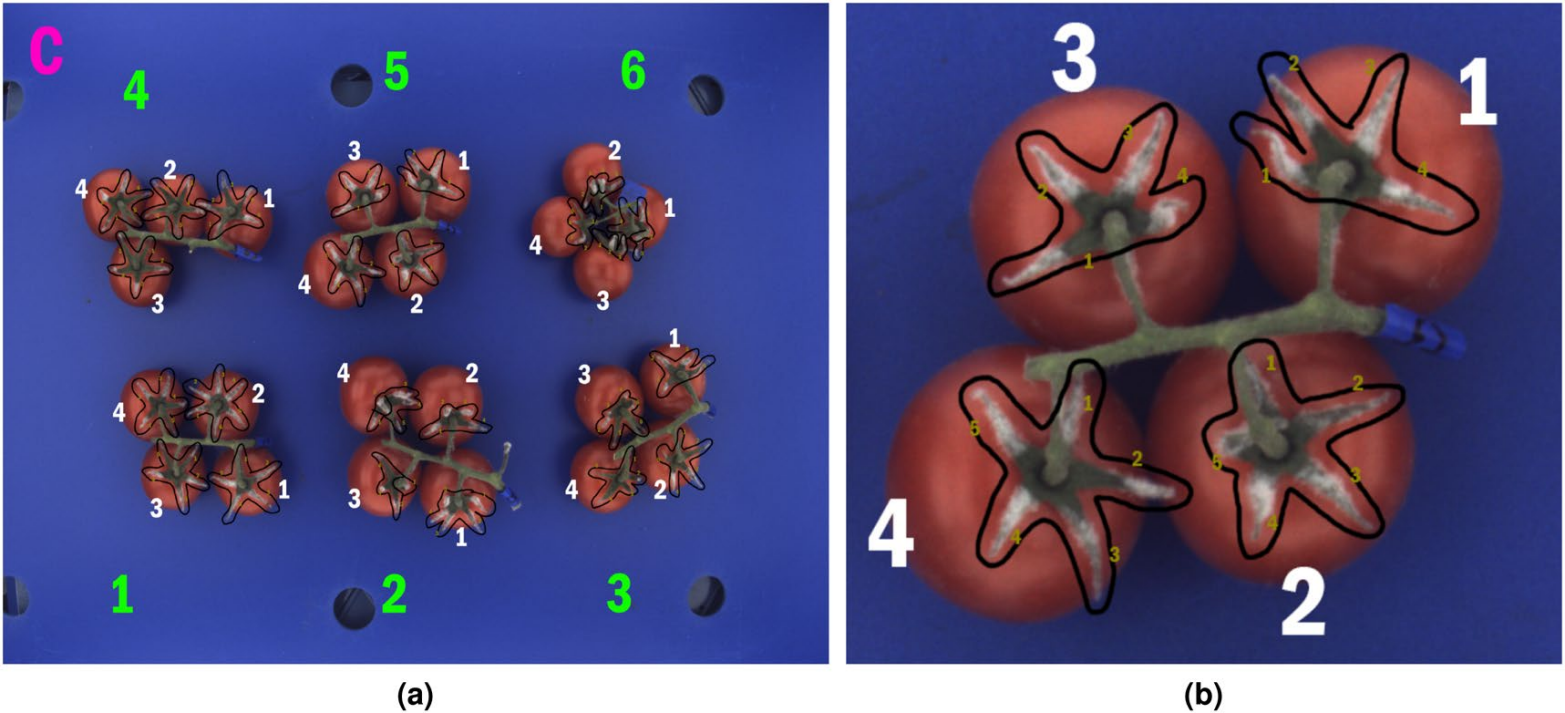
(**a**) Color cabinet RGB image of Batch C with marks used for enumeration. For enumeration of color cabinet image, an alphabet label was used for denoting the batch i.e. letter from A to F, while tomato trusses were enumerated from 1 to 6. Tomatoes within trusses were numbered from 1 to either 4 or 5, depending on the number of tomatoes in a truss. Sepals are denoted with numbers from 1 up to either 4 or 5, depending on the total number of sepals that are visible in the image. The sepals numbering was made according to the orientation rules presented in Fig. 1. (**b**) Cropped region in color cabinet image on the left related to the truss 5.

The color images were used as ground truth for the assessment of the degree of fungal infection. Figure 3 shows a color image of trusses for Batch C and the encoding rules which were adopted to relate the assessed fungi infection level of sepals with their hyperspectral responses. According to the encoding rules, each sepal was assigned an identifier that is a 4-digit number where digits correspond to batch label, number of truss, tomato and sepal, respectively.

In summary, two datasets were created in this experiment: one of hyperspectral data before any visible evidence of fungus, and another one of the RGB images after the fungal infection has materialized.

### Ground truth collection

Assessment of fungi infection severity levels was conducted by a panel of researchers. Obtaining the diagnosis on the levels of sepals’ infection was a joint work of 11 researchers with diverse expertise including remote sensing, image processing, data science and agriculture. Diagnosis was expressed as a range between 0 and 5, where 0 denoted no infection and 5 a severe infection which has spread across the complete sepal. Visual grading was performed in a single day by presenting RGB images of tomatoes from the last day of the experiment to the panel. Each sepal on RGB images was magnified and experts independently graded the infection level. Fusion of diagnoses was performed using principal component analysis (PCA) where the first PCA component explained 98% of the variability in the infection grading. The final infection severity of each sepal is then computed by projecting the infection grades from 11 researchers onto the first component. Summary statistic of sepals’ diagnoses across batches is provided in Table 2. All batches contain similar numbers of sepals: 105, 116, 108, 117, 114, 110. Table includes corresponding mean, standard deviation, coefficient of variation, as well as percentages of sepals with low, moderate and high infection, where low denotes sepals with diagnosis less than 1, and high more than 4. Derived statistics show that sepals from batch F have low infection level, but highest relative level of dispersion around the mean. Sepals from batch E have also lower infection levels compared to batches A, B, C and D.

**Table 2:** A summary statistics of diagnosis of sepals across the 6 batches of tomatoes used in the experiments.

Batch	Mean	Standard deviation	Coeff. of variation	Low infection	Moderate infection	High infection
A	2.26	1.23	0.54	20.00%	71.43%	8.57%
B	2.77	1.60	0.58	21.56%	49.13%	29.31%
C	2.68	1.65	0.61	24.07%	48.15%	27.78%
D	2.44	1.69	0.69	33.33%	42.74%	23.93%
E	1.14	1.02	0.89	50.87%	44.74%	4.39%
F	0.10	0.26	2.54	99.09%	0.91%	0.00%

### Identification of fungal strain

**Figure 4:**
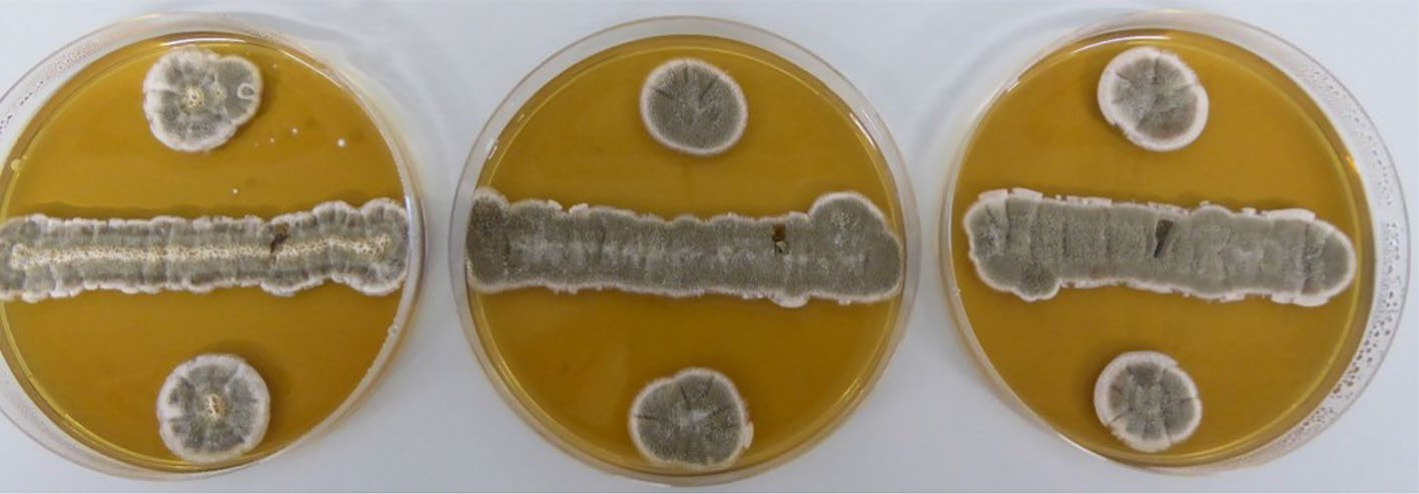
Fungal growth of spores collected from a random tomato calyx on Malt Extract Agar plates.

Three samples from sepals from a randomly selected truss were collected and analyzed for identification of the fungal strain. A microbiologist at WFBR incubated the samples on Malt Extract Agar plates for 5 days at 22 °C, to visually classify the strains (see Fig. 4). The visual observation indicated that the three samples likely belonged to one strain, which could either be *Penicillium sp.* or *Cladosporium sp.* Further identification was not carried out due to an unforeseen high temperature in the incubator, which destroyed the samples before further identification.

### Software

The preproceesing pipeline is implemented in Python v3.6 using the following open-source libraries: Spectral Python (SPy)^42^ for reading hyperspectral data, Scikit-Image (skimage)^43^ for noise removal within spectral responses and OpenCV^44^ for image manipulation during calyx and stem segmentation. Machine learning and statistics pipelines also also implemented in Python using the Scikit-learn (sklearn)^45^, XGBoost^46^ and SHAP^47^ libraries.

## Hyperspectral data processing

Although the aforementioned datasets contain complete information pertaining to the tomatoes, the key interest is in the information content of the sepals, which is where the fungal infection manifests. The following subsections outline the process of data cleaning and preprocessing for extracting the content corresponding to the sepals.

### Radiometric correction

Analysis begins with a flat-field correction of reflectance measurements and noise reduction for each spectral band. Denoting hyperspectral image as a three order tensor ${\mathbf {m}} \in \mathbb {R}^{M \times N \times S}$ where *M* and *N* are the numbers of rows and columns respectively, while *S* denotes the number of selected spectral bands. Flat-field correction was done with the standard approach used in^48,49^ where a reflectance ${\mathbf {m}}_{\mathrm {c}}(i,j,\lambda )$ per pixel (*i*, *j*) and for each spectral band $\lambda \in \mathbb {R}$ is obtained as follows:

1 $$\begin{aligned} {\mathbf {m}}_{\mathrm {c}}(i,j,\lambda ) = \frac{{\mathbf {m}}(i,j,\lambda ) - {\mathbf {b}}(\lambda )}{{\mathbf {w}}(\lambda ) - {\mathbf {b}}(\lambda )} \end{aligned}$$

where ${\mathbf {m}}(i,j,\lambda )$ represents the intensity of the measured hyperspectral response of the observed pixel in the image, and ${\mathbf {b}}(\lambda )$ and ${\mathbf {w}}(\lambda )$ are the hyperspectral responses of the *black* and *white* references respectively. After applying (1) over all the spectral pixels and across all available wavelengths $\lambda$, a noise removal step is conducted on images for each $\lambda$ separately.

### Noise removal

**Figure 5:**
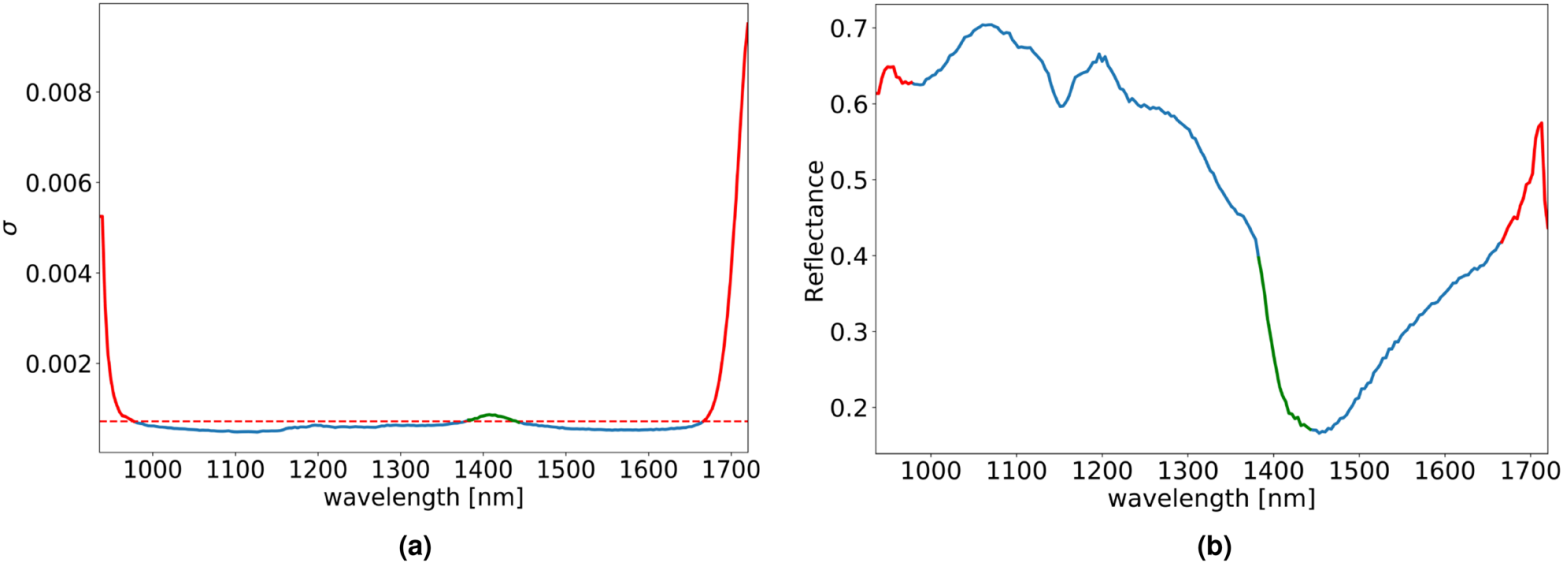
(**a**) Estimated $\sigma$ through spectral bands with a dashed red line denoting the adopted threshold for selection of relevant spectral bands. $\sigma$s values are colored blue/red color to specify if the corresponding spectral bands are kept or disregarded from further analysis. The spectral bands which correspond to the $\sigma$s colored in green are kept since they provide useful information for sepal segmentation (**b**) Spectral response of a pixel belonging to a sepal marked with blue, red and green colors for correspondence with the estimated $\sigma$s in the graph on the left.

On spectral bands of the hyperspectral image, noise standard deviation $\sigma$ is estimated based on the median absolute deviation of the wavelet detail coefficients as described in “Model explanation” section of^50^. In Fig. 5, with red and green color are denoted $\sigma$s which have noise level greater than threshold set to 1.5 times the minimum estimated $\sigma$. The spectral bands corresponding to the $\sigma$s marked in red are discarded from further analysis. Although the $\sigma$s coloured in green have greater values than the adopted threshold, the corresponding spectral bands are not discarded because of spectral and spatial information useful for sepal segmentation.

Overall, the first 13 and last 16 spectral bands were rejected, resulting in 194 spectral bands in the range $\lambda = [980~{\text{nm}}, 1660~{\text{nm}}]$ for further processing. For noise reduction, a wavelet soft-thresholding procedure^51^ is applied on these bands. Notice that the noise removal is applied as a preprocessing step to separate the tomato region from the calyx and stem. This step is omitted when extracting the hyperspectral responses of the sepal tips (see subsection Spectral features), since the region of the tips is tiny and any noise removal can pollute the reflectance at the tip with the reflectance of the background.

This procedure identifies the noisy spectral bands, but doesn’t take the noise present between neighboring spectral bands into account. In the next step, this inter-band noise, caused by the limited spectral resolution of the device, is addressed. Application of a 1D median filter to the spectra at each pixel provides a much smoother reflectance while suppressing the noise between consecutive spectral bands.

### Calyx and stem segmentation

**Figure 6:**
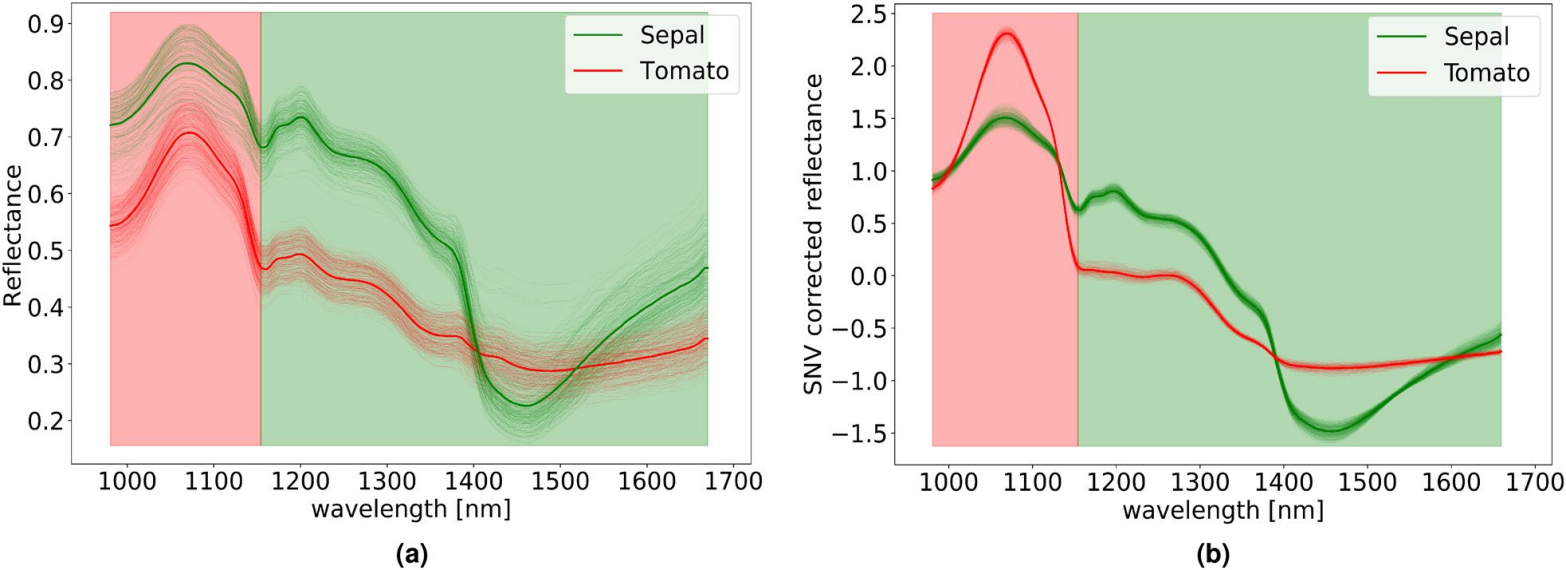
(**a**) Spectral responses of pixels within the tomato and sepal regions and their corresponding mean responses. (**b**) Spectral response after SNV transformation. Shaded regions in the graphs denote which spectral bands are used for the creation of masks for tomato (red region) and calyx-stem (green region).

After noise removal, calyx and stem segmentation is performed by manipulating the spectral bands. Figure 6a displays the reflectance of pixels that belong to the tomato and sepal regions. The variation in the responses of reflectance up to 1150 nm is higher for the tomato region, when compared against the variance of the sepal region for the same spectral range. From 1150 nm onwards, the situation is reversed and the variation in reflectance in the sepal region is higher. Therefore, $\lambda _{\mathrm {d}} = 1150~{\text{nm}}$ is selected as a threshold wavelength which divides spectral range into two regions, one preceding $\lambda _{\mathrm {d}}$ is used for generating mask for the tomatoes and the one proceeding $\lambda _{\mathrm {d}}$ is used for obtaining the calyx and stem mask. Next, a per-spectrum basis preprocessing step known as standard normal variate transformation (SNV)^52,53^ is applied. SNV is applied only to the hyperspectral responses in the selected range of wavelengths defined in the subsection Noise removal. SNV transformation suppresses the possible large amount of variability among hyperspectral responses caused due to scattering effects within the same region in the image^31,38,54,55^ (see Fig. 6b).

To extract the variation of the reflectance for each pixel in the image, 1D convolution is applied to the image ${\mathbf {m}}_{\mathrm {c}}$ with a first-order derivative of 1D Gaussian function ${\mathbf {g}}(x)$:

2 $$\begin{aligned} {\mathbf {g}}^{\prime }(x) = -\frac{x}{\sigma _{\mathrm {g}}^{2}}{\mathbf {g}}(x) \end{aligned}$$

where ${\mathbf {g}}(x)=\frac{1}{\sqrt{2\pi }\sigma _{\mathrm {g}}}e^{-\frac{x^{2}}{2\sigma _{\mathrm {g}}^{2}}}$ and $\sigma _{\mathrm {g}}$ indicates the filter width. Motivation for the use of ${\mathbf {g}}^{\prime }$ came from^56^ where ${\mathbf {g}}^{\prime }$ was used as an approximation for the optimal step edge operator. Convolution of ${\mathbf {m}}_{\mathrm {c}}$ with ${\mathbf {g}}^{\prime }$ can be expressed as:

3 $$\begin{aligned} ({\mathbf {m}}_{\mathrm {c}} *{\mathbf {g}}^{\prime })(i,j,\lambda _{\mathrm {n}}) = \sum _{\lambda _{\mathrm {s}} = -L(\sigma _{\mathrm {g}})}^{L(\sigma _{\mathrm {g}})} \Big ({\mathbf {m}}_{\mathrm {c}}(i,j,\lambda _{\mathrm {n}}) - {\mathbf {m}}_{\mathrm {c}}(i,j,\lambda _{\mathrm {s}})\Big ) {\mathbf {g}}^{\prime }(\lambda _{\mathrm {s}}) \end{aligned}$$

where filter width is defined as $L(\sigma _{\mathrm {g}})$ which depends on the choice of $\sigma _{\mathrm {g}}$.

**Figure 7:**
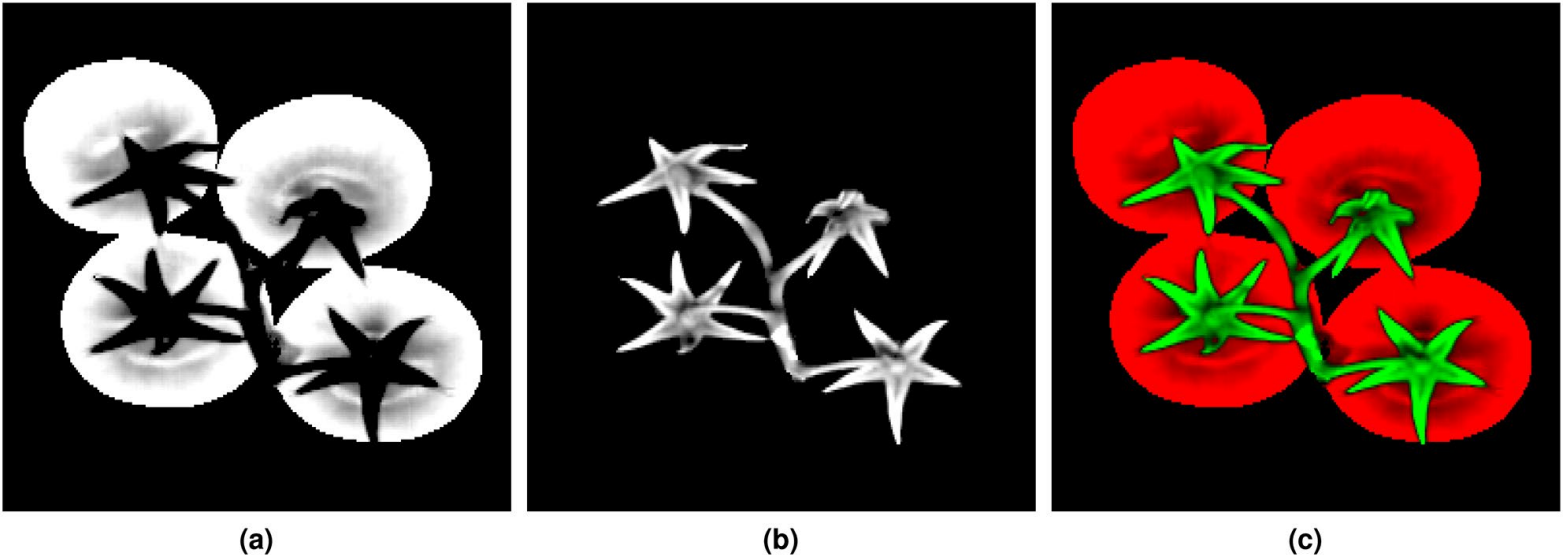
(**a**) *Significance* masks for tomato (**b**) calyx-stem region and (**c**) pseudo color image created from them.

The convoluted spectral image can now be used to generate two maps of *significance* which indicate for each pixel position its association to the tomato or calyx-stem region. Denoting results of convolution as ${\mathbf {r}}(i,j,n) = ({\mathbf {m}}_{\mathrm {c}} *{\mathbf {g}}^{\prime })(i,j,\lambda _{\mathrm {n}})$ then significance masks ${\mathbf {t}},{\mathbf {s}}$ for tomato and calyx-stem regions respectively are generated in the following way for each pixel position (*i*, *j*):

4 $$\begin{aligned} \begin{aligned} {\mathbf {t}}(i,j)&= \sum _{n = \lambda _{\mathrm {1}}}^{\lambda _{\mathrm {d}}} \left| {\mathbf {r}}(i,j,n)\right| \\ {\mathbf {s}}(i,j)&= \sum _{n = \lambda _{\mathrm {d}}}^{\lambda _{\mathrm {N}}} \left| {\mathbf {r}}(i,j,n) \right| \end{aligned} \end{aligned}$$

where $\lambda _{\mathrm {1}} = 980~{\text{nm}}$ and $\lambda _{\mathrm {N}} = 1660~{\text{nm}}$ are the first and last spectral bands of the selected spectral range. An example of *significance* maps is shown in Fig. 7.

**Figure 8:**
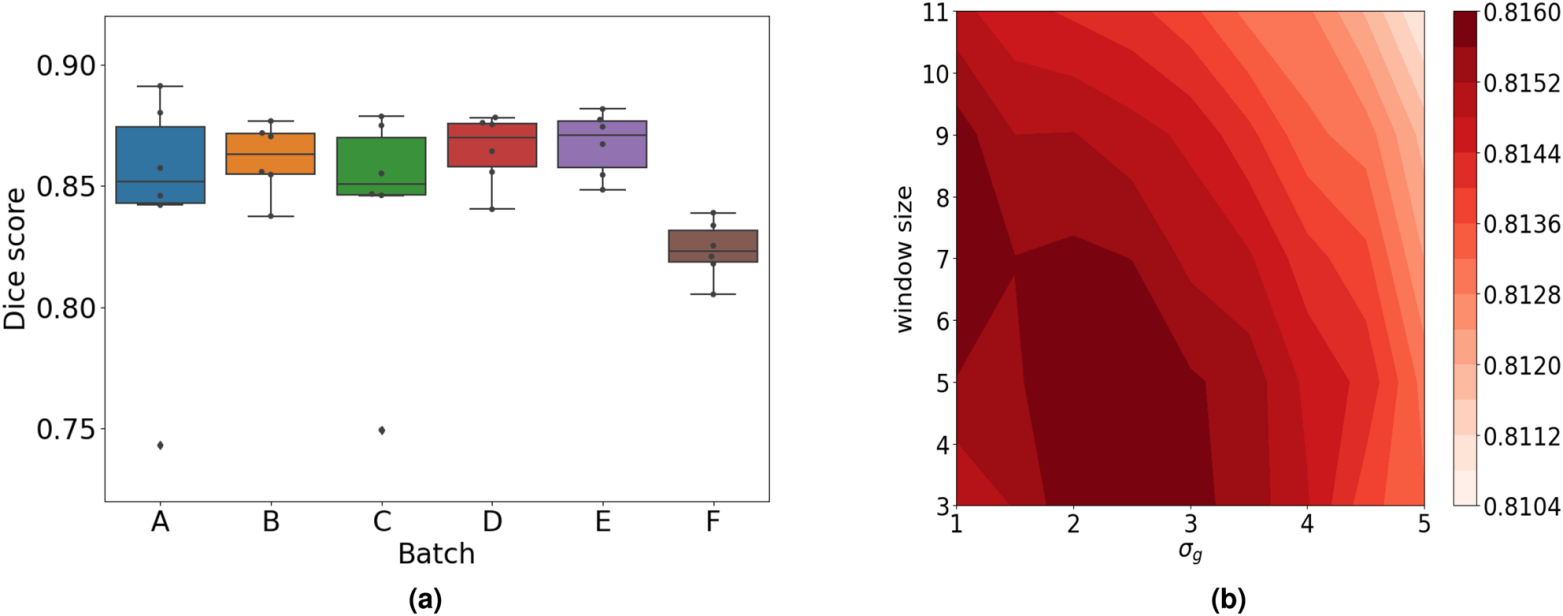
(**a**) Obtained Dice scores per batches with the following values for parameters: windows size for median filter equal to 5 and $\sigma _{g} = 2.5$ (**b**) Averaged Dice scores during grid search for parameters $\sigma _{g}$ and windows size for median filter.

The intensity of the calculated maps is then rescaled using percentiles such that the border between calyx-stem and tomato region is amplified. Final binary maps for the region of tomato and calyx-stem are obtained after application of the Otsu method^57^ on normalized significance maps ${\mathbf {t}}$ and ${\mathbf {s}}$. Using this procedure, the spectral image can be automatically segmented into the tomato and calyx-stem regions. The precision and accuracy of the automatic segmentation of calyx-stem region is evaluated by comparing its estimated mask with manually created ground truth mask. Figure 8 shows results of comparison using Sørensen Dice coefficient metric (also called Dice score)^58,59^.

### Sepal extraction

**Figure 9:**
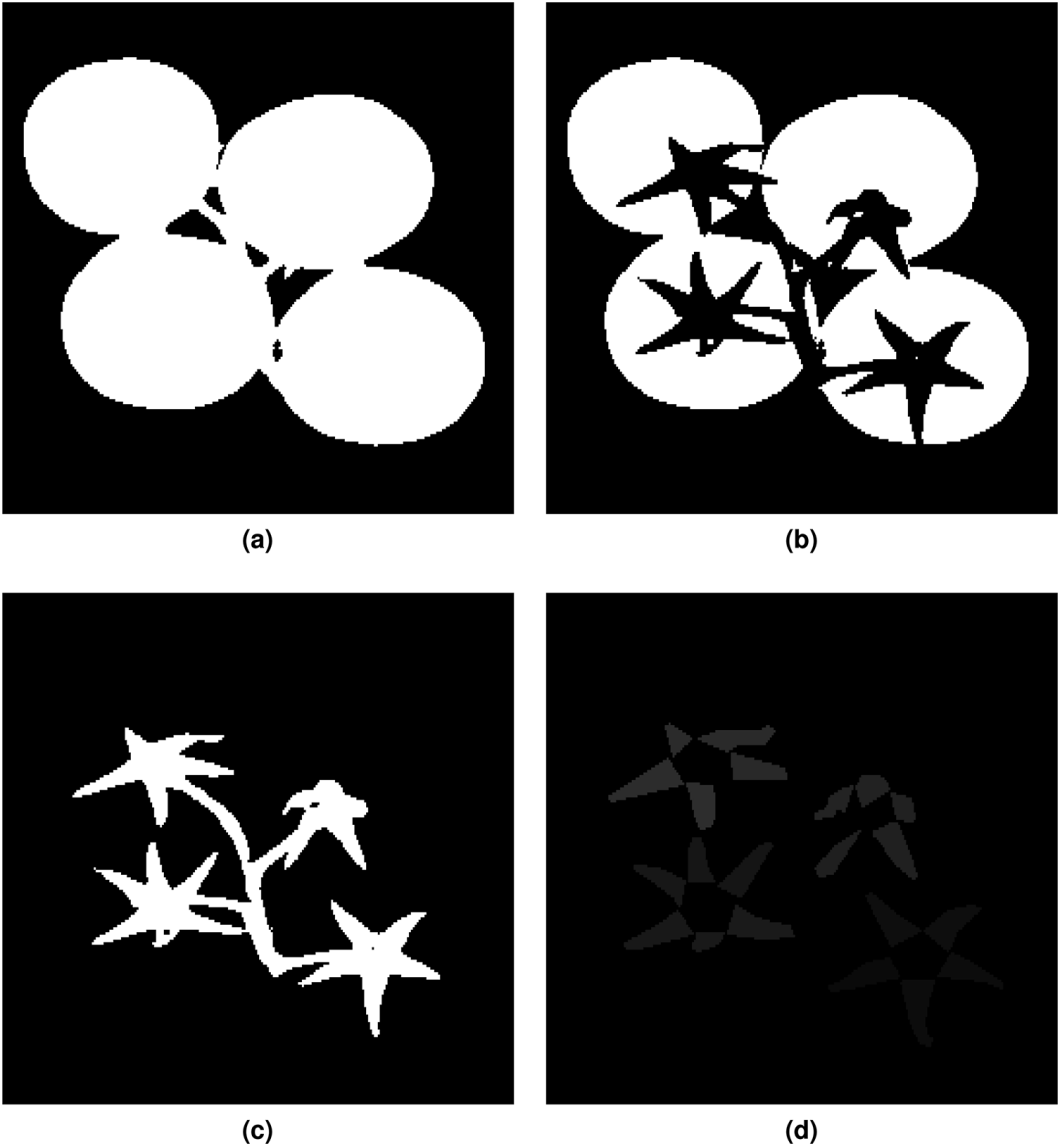
(**a**) Mask of the truss, (**b**) mask of the region belonging to tomatoes, (**c**) mask of the ROI (sepal region) and (**d**) sepal identification mask.

Next preprocessing stage is to extract the pixels corresponding only to the sepals from the hyperspectral images. This is a semi-automated procedure that is conducted in three steps:

- The first step requires segmentation of calyx-stem region of interest (ROI), as discussed in the previous section (see Fig. 9 under the labels a, b and c);
- The second step consists of manual creation of a “rough” sepal identification mask for each truss (see Fig. 9d);
- The final step involves an intersection on the ROI with the manual masks. This step provides the mask for a sepal belonging to a particular tomato.

### Spectral features

The preprocessing steps described in the previous section lead to the extraction of spectra corresponding to individual sepals belonging to each tomato. Each sepal pixel has its spectral signature across 194 wavelength bands, having a good signal-to-noise ratio. Information on the level of pixels is aggregated to obtained features on the level of sepals. The mean and standard deviation of pixel values, corresponding to sepal for each of the 194 wavelength bands, is then extracted. Finally, each sepal is described by two 194 sized feature vectors—the mean spectra and the spectral standard deviation.

## Results

### Machine learning based predictive models

Two ensemble methods, Random Forest^60^ and extreme gradient boosting (XGBoost)^61^, were used to train the predictive models for the assessment of fungi infection severity. Both are able to deal with a large number of features and are not vulnerable to overfitting. Random Forest algorithm builds many decision trees independently and then averages their predictions. XGBoost uses feature sampling, same as Random Forest, but builds trees through an iterative process. In each iteration, trees are added to correct the errors made by the existing ones.

Training predictive models, based on Random Forest and XGBoost algorithms, included training/test split into 80% and 20% of data and hyperparameter tuning with cross-validation procedures on training set. 10-fold as well as group-fold cross-validation methods were used to evaluate the performance of the models and select parameters of the models. Group-fold cross-validation ensures that the same group is not represented in both training and test sets. In the reported experiments, groups correspond to different tomato batches. Cross-validation results were obtained as the best over grid search procedure for parameters with objective function to maximize R2 score of the model. Optimal parameters are further used to fit model on the whole training data and final validation is performed on the test set. Table 3 summarizes the results for both models and both types of cross-validation methods, as well as the results on hold-out test set. Numbers represent mean values of 5 experiments with random training/test splits. Besides R2 score, other metrics - Root Mean Squared Error (RMSE) and Pearson correlation, were also used to inspect the performance of the models.

**Table 3:** Performance of the models.

Model	Validation	RMSE	R2 score	Pearson corr.
Random Forest	10-Fold cross validation	0.947	0.673	0.820
XGBoost	10-Fold cross validation	0.976	0.653	0.809
Random Forest	Group-K-Fold validation	1.077	0.572	0.757
XGBoost	Group-K-Fold validation	1.132	0.528	0.731
Random Forest	Test; param. from 10-Fold CV	0.918	0.674	0.827
XGBoost	Test; param. from 10-Fold CV	0.932	0.659	0.818
Random Forest	Test; param. from Group-K-Fold	0.905	0.699	0.837
XGBoost	Test; param. from Group-K-Fold	1.016	0.606	0.791

**Figure 10:**
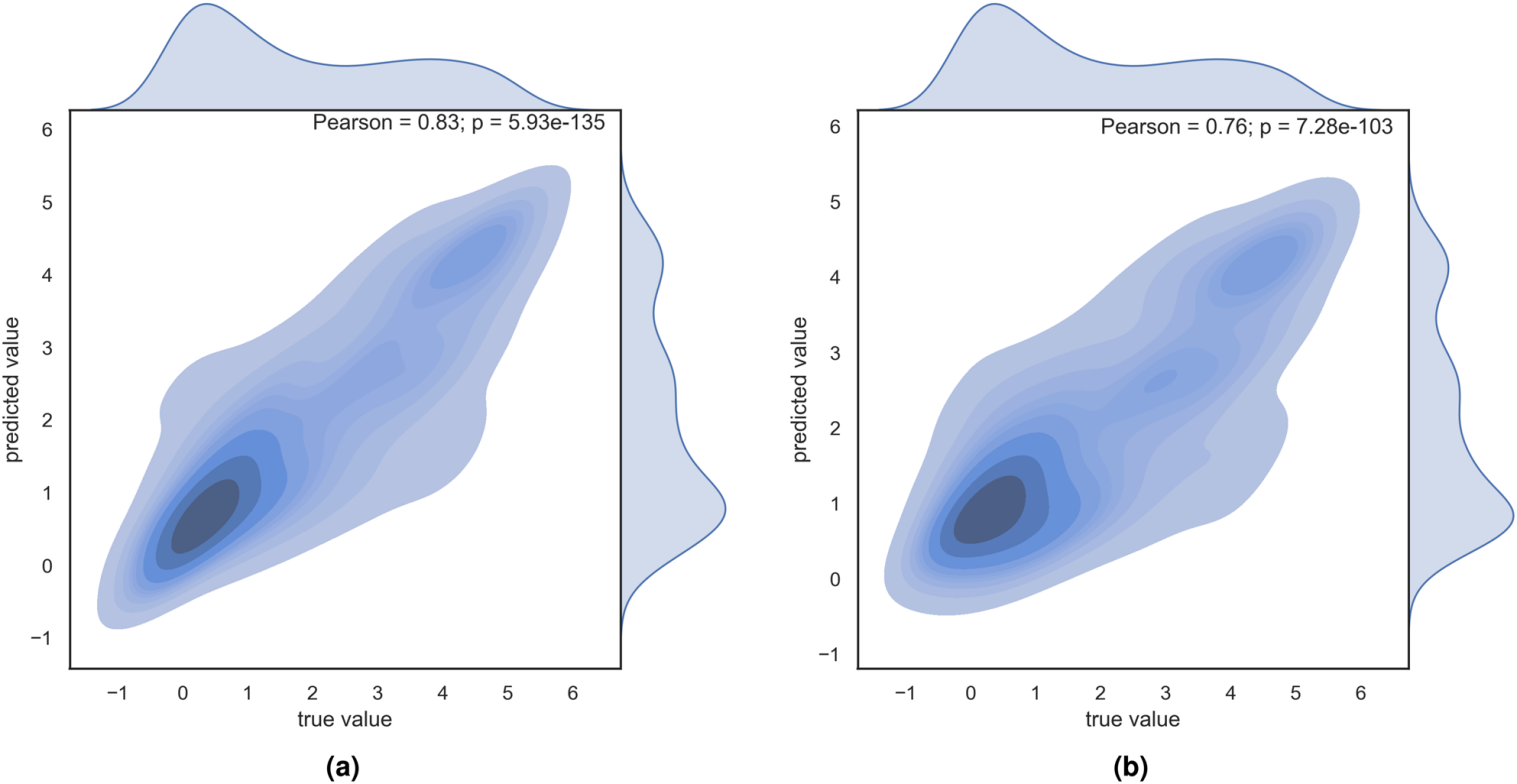
Joint plot of predicted and true values with marginal distributions (**a**) 10-fold, (**b**) Group-K-fold.

Random Forest models provided slightly better results. Predicted fungal infection severity correlates well with the ground truth estimates with Pearson correlation of 0.820 and 0.757, and a high proportion of the variance explained with R2 scores of 0.673 and 0.572 for 10-fold cross validation and group cross validation, respectively. Similarly, Random Forest models had lower errors expressed through RMSE compared to the XGBoost. Slightly lower performance of group-k-fold validation comes from smaller number of training samples in folds split according to 6 batches and the intrinsic variability of different tomatoes coming from different batches. Thus, group-k-fold results provide estimates on the lower bound of the model’s performance which could be further improved with more samples from different origins. Figure 10 presents joint plots of true and predicted observations in the form of kernel-density distribution and includes the marginal distribution of true and predicted sensitivity of tomato sepals to fungal infection. Performance on the test set results did not drop and remained stable across 5 repetitions of the experiments.

**Figure 11:**
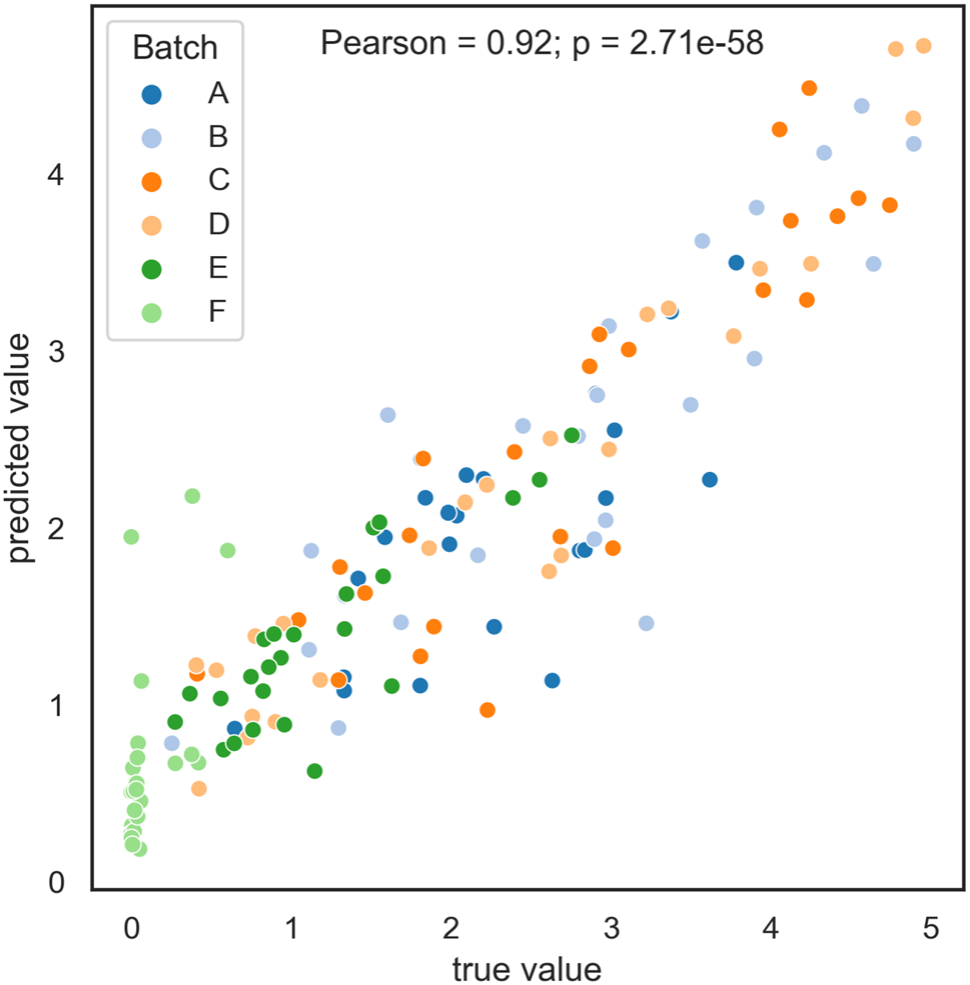
Aggregated true and predicted values at tomato level.

From an application perspective, the key question is the susceptibility to fungal infections of individual tomatoes, as well as specific batches, rather than individual sepals. To aggregate predictions from sepal to tomato level all predicted sepal values were utilized - obtained through cross-validation and on test set. For each tomato, the mean prediction is computed over all of its sepals as the final tomato susceptibility. The true tomato susceptibility is also calculated in the same manner. Tomato level predictions are plotted against the true predictions in the scatter plot presented in Fig. 11. The overall predictive performance remains high at tomato level. The plot also unveils information on batch specific sensitivity to infection, with a Pearson correlation of 0.92. It can also be observed that the predictions affirm what is already known from the ground truth—Batch F, distinct for being a new crop (the first harvest) was almost resilient to the infection, and batches A and E showed less sensitivity to the infection compared to the other three batches.

### Model explanation

In this section, focus is on interpreting the developed model. Interpretability, here, refers to the understanding of which features are most useful in a model’s prediction. Such information has practical advantages, for example, it could help in selecting a limited number of wavelengths to construct a multi-spectral camera specific to the problem addressed in this paper.

Ensemble methods based on decision trees, like Random Forest and XGBboost, offer global interpretations of input features which can help in understanding the impact/importance of each feature to model predictions^62^. Although highly useful, such interpretations have the drawback that they do not allow local interpretation, that is, the impact of input features on prediction over individual samples^63^.

In the next subsections, two model explanation approaches are explored to interpret the model results globally as well as locally.

#### Global interpretation

**Figure 12:**
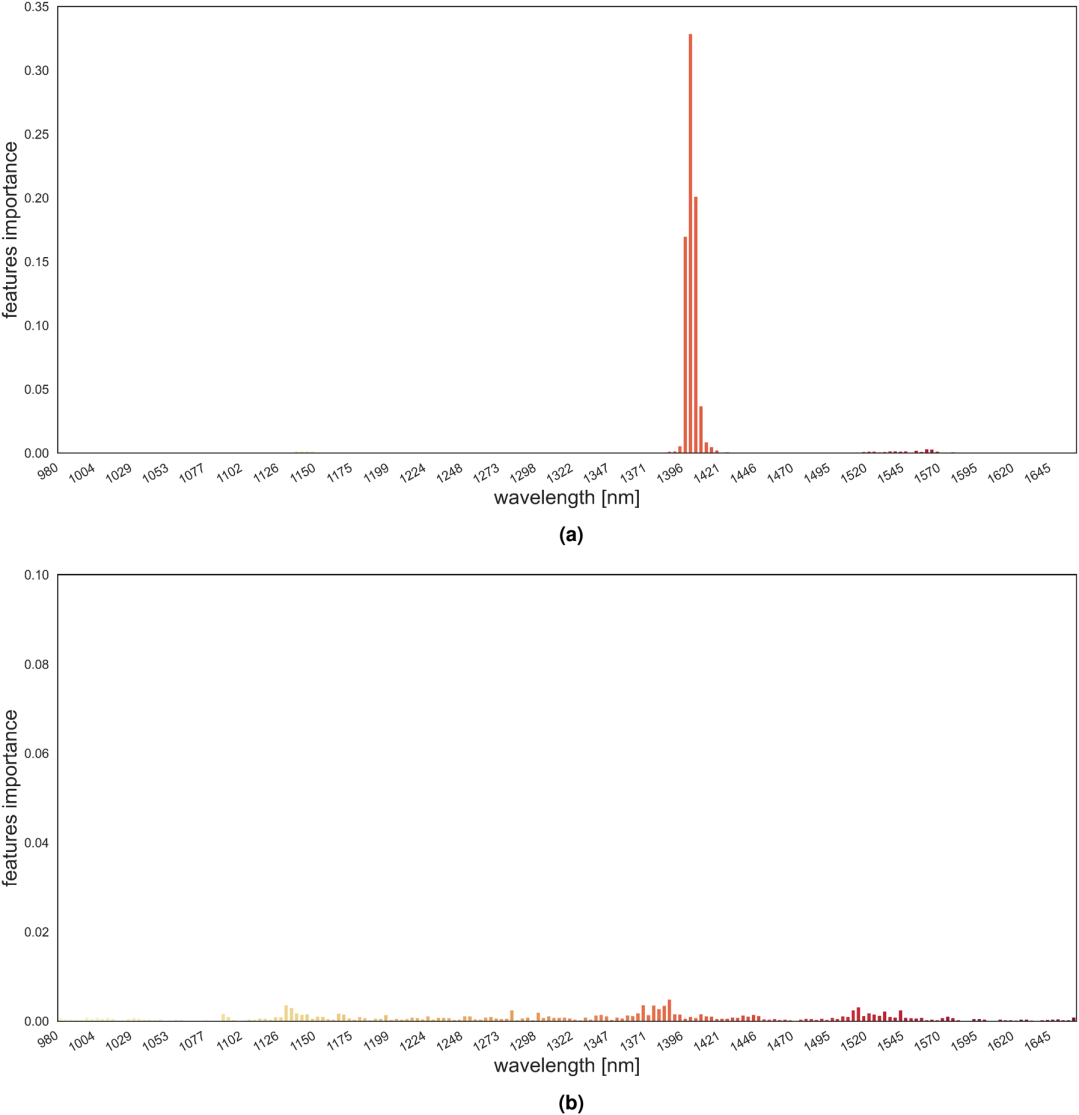
Hyperspectral wavelengths importance: (**a**) features corresponding to the mean values, (**b**) features corresponding to the standard deviation values.

Not all features contribute equally to the model decisions. In other words, not all wavelengths are equally relevant for making the decision on potential fungal severity. Global feature importance analysis derived from the best performing model, based on Random Forest method, is presented in Fig. 12.

Results indicate that the majority of the examined spectral wavelengths are not important for the prediction of future fungal development. What clearly stands out as significant is the range between 1390 nm and 1420 nm. Furthermore, features representing sepals’ mean have higher importance compared to standard deviation features. In summary, according to the feature importance values, the mean spectra feature in the range 1390–1420 nm contribute most to the model’s performance.

When predictive models were built only on this range, model performance either slightly diminished compared to the model trained on the whole examined spectral range. Model achieved R2 scores of 0.608, Pearson correlation of 0.781 and RMSE if 1.025 in 10-fold cross validation experiment and on test set 0.656, 0.812 and 0.991 for R2, Pearson correlation and RMSE metrics.

**Figure 13:**
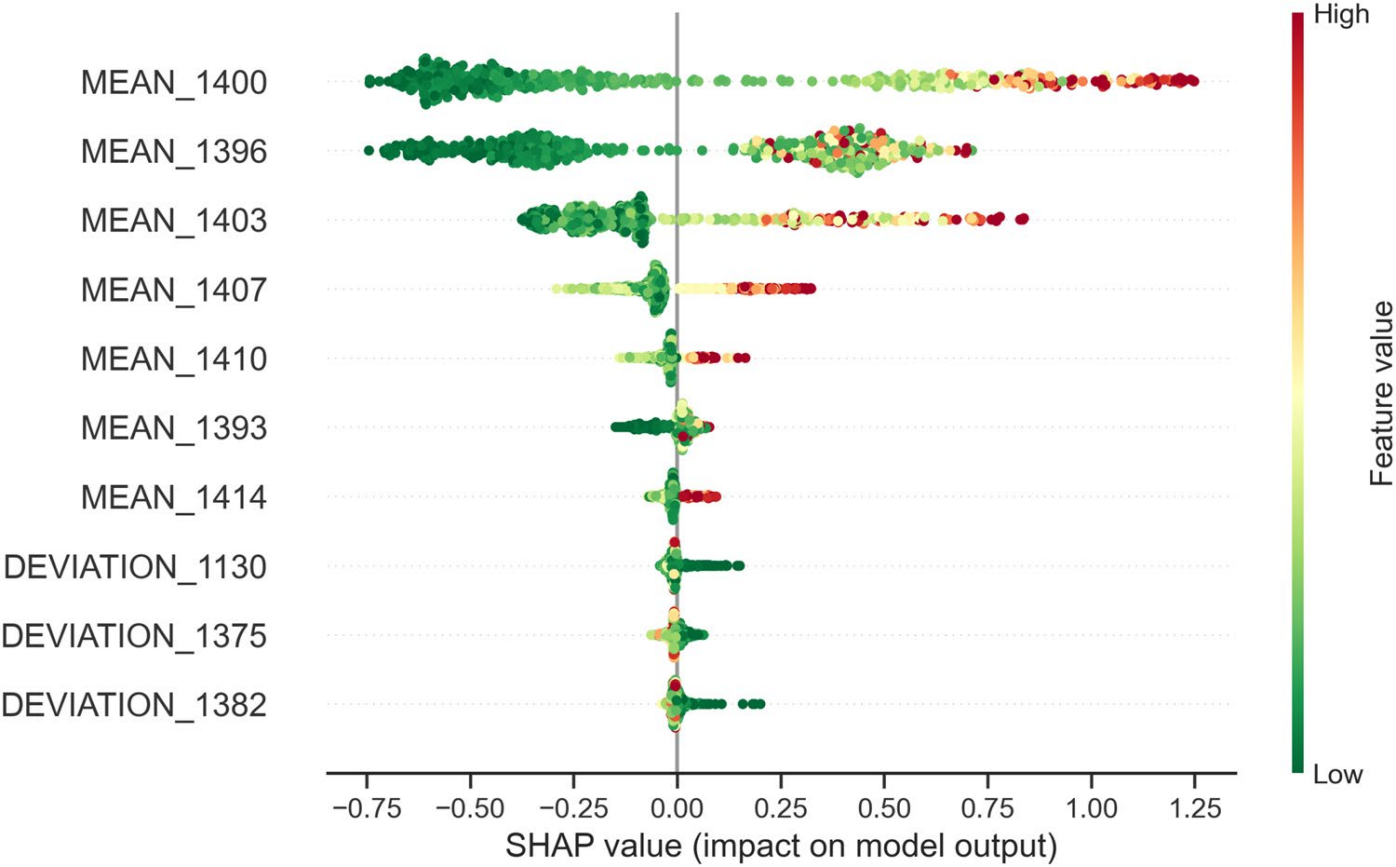
The 10 most important features, listed from top to bottom, and their contribution. Dots visualized in each row, corresponding to a specific feature, represent feature values of all the training samples.

For further understanding of the trained model, a unified framework SHAP (SHapley Additive exPlanations)^63,64^ is used for interpreting the model and its predictions. SHAP framework calculates Shapley value for each feature based on a conditional expectation function that assigns its contribution to a particular prediction. Prediction is explained through additive features’ importance, i.e. the contribution is fairly distributed among the features according to the calculated conditional expectations. On a global scale, this analysis estimates the effect of how each feature contributes to the classification decision and thus provides a full model explanation. Contributions can be positive or negative when compared to the baseline, which is the average model prediction. Summary plots for 10 top-ranking features derived from SHAP framework are provided in Fig. 13.

It can be observed that the main top-ranking features (mainly top 5) correspond to sepals’ mean spectral response in the range from 1390–1410 nm. This result agrees with the global interpretation from the ensemble feature importance metric. All features exhibit a similar pattern - if the feature value is high, so is its SHAP value. That is, sepals with high values of these features are more susceptible to higher infection incidence. The smaller feature values in this range correspond to lower infection susceptibility. Among 10 top-ranking features, 3 correspond to sepal’s standard deviation features, but their contribution is significantly low in final model prediction.

#### Instance level model interpretation

**Figure 14:**
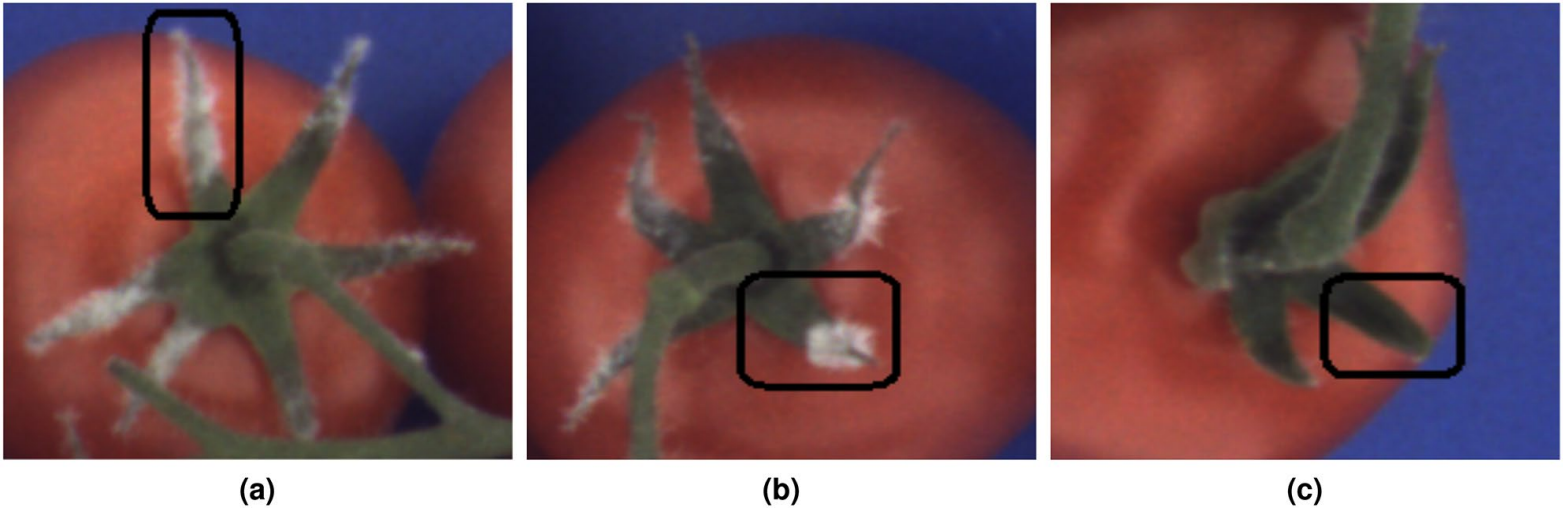
Examples of sepals with different levels of infection: (**a**) severe infection, (**b**) moderate infection, (**c**) no infection.

In addition to global explanation, SHAP also enables interpretation of model predictions over individual samples. For three sepals from the test set, with high, moderate and no infection, corresponding explanations are provided. Sepals with different infection levels are marked in Fig. 14. Model predictions for the first, second and third sepal are 4.17, 2.16 and 0.48, respectively. The predictions are close to the grades assigned by the panel of researchers that are 4.54, 2.89 and zero.

**Figure 15:**
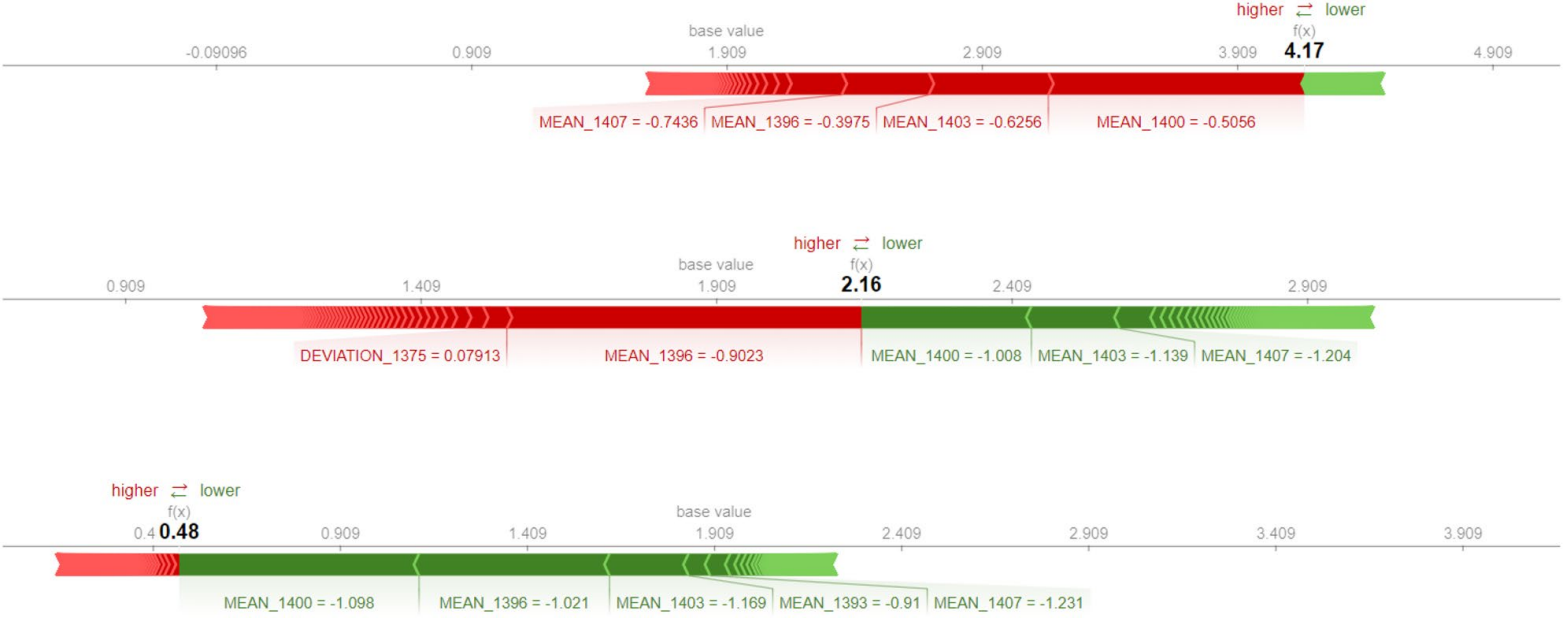
Instance explanations for three sepals from Fig. 14. Red colored features are pushing the prediction higher, while the green ones are nudging the prediction lower.

Explanations for the predicted sensitivity to infection for selected sepals are shown in Fig. 15. These instance level explanations uncover which features and their particular values contributed to the final prediction for that specific sepal. Baseline prediction is the mean value observed in the ground truth data.

In case of the first sepal, one can observe that most of the features contributed in the direction of high infection, as indicated by red arrows. Higher values of features $MEAN\_1400$, $MEAN\_1396$, $MEAN\_1403$ and $MEAN\_1407$ imply high sensitivity to infection. From the explanation graph for the second sepal, there is a decrease in the contribution of red features and an increase of green features that lower the value of prediction. The obtained explanation also aligns well with infection severity estimated by the panel of researchers. The predicted value for the third sepal, on which the development of the infection is not observed, is quite low at 0.40 and lines up with the true value. Related explanation confirms the high contribution of features in the direction of lower infection, as denoted with green arrows. The highest contribution in this prediction comes from features $MEAN\_1396$, $MEAN\_1400$ and $MEAN\_1403$. Overall, the instance level explanations for the three scenarios are consistent with the global explanations. Small differences in baseline predictions in presented examples come from estimates of the means in different cross validation data folds.

## Discussion

**Figure 16:**
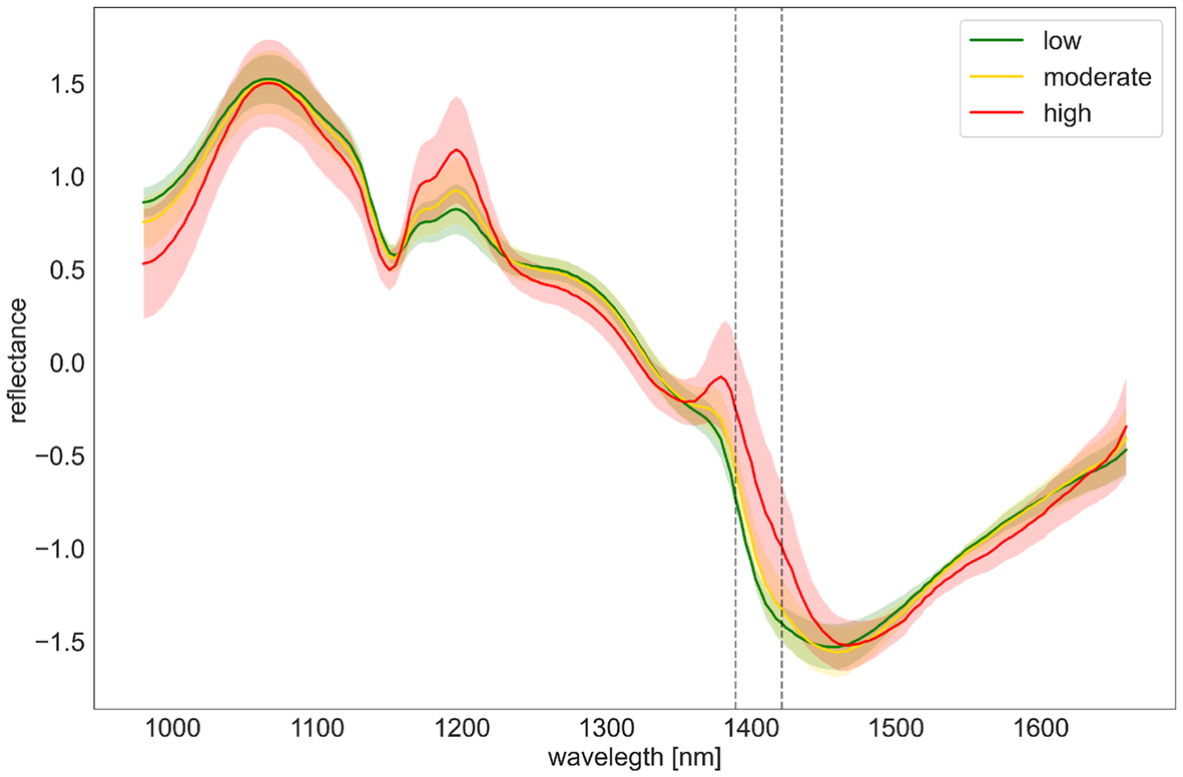
Mean spectral response with with standard deviations of sepals grouped into 3 categories. Two vertical lines highlight the range of 1390–1420 that model explanation identified as the most important.

The model explanations discussed above are valuable to understand which features/wavelengths contribute the most in predicting the susceptibility of sepals to future fungal infection. In particular, from both global and local interpretations, the range 1390 nm to 1420 nm stands out as a key indicator in predicting future sepal susceptibility. This is likely not coincidental. As can be noticed in Fig.16, there is a sharp drop in spectral reflectance in this range. Research reported in this paper does not make an explicit correlation between the NIR spectral response and the actual compounds responsible for the absorption/reflection. However, some insights can be derived from the literature.

NIR reflectance is due to the vibration overtones and combination modes attributed to functional groups containing a hydrogen atom^65^. Drawing parallels from the literature on fruits and leaves, the NIR region identified in this paper corresponds mainly to one of the typical water absorption regions (i.e. spectrum with vibration overtone of the O–H bonds)^66–70^. It is well known that, in addition to the O–H bonds, NIR absorption features are also due to stretching and bending vibrations of C–H, N–H, C–O within organic compounds such as, lignin, cellulose, starch, proteins and nitrogen^66,70^. However, the absorption of these compounds is not strong and is cloaked in presence of water content in leaves^70^. In drier leaves, the absorption features of other compounds are expected to be more clear.

For healthy sepals, which have a higher water content, one interpretation is that the presence of O-H bond in water leads to stronger absorption (i.e. lower reflectance) of NIR in this range. As discussed, it can be assumed that the effects of other compounds on healthy sepals are masked due to high water content. Machine learning based model explanations also demonstrated that lower values of mean features correspond to reduced susceptibility to fungal infection. Figure 16 presents mean spectral responses with standard deviations of sepals grouped into 3 categories, where red, yellow and green denote sepals with severe (> 4), moderate (1–4) and no infection respectively (<1). Overall, highly susceptible sepals have markedly different spectral responses in comparison to the healthy ones, and the spectra of moderately susceptible sepals lie in-between the extremes.

From a physiological point of view, a low water content of the sepal tissue may indicate that the tissue is not fully hydrated or may have limited capacity to attract and retain water. The latter may be related to a lack of sufficient osmotic compounds (such as sugars) or to an impaired cell membrane integrity and/or the presence of dead cells. Such conditions likely make the tissue more vulnerable to fungal infections. The lack of any infections in the trusses harvested from the new crop, Batch F, that was grown under supplemental lighting, shows that the state of the crop is also an important determinant for the physiological features of the sepals. Moreover, this is a potential reason why the model does not perform equally well on this batch. For measuring traits of fruits and vegetables, NIR models are known to perform poorly when used on batches that are markedly different from the ones used in model construction^71–73^. Although batch F samples were used in training, it is clearly different from the rest, with different growth conditions, this being the first crop, and the infection severity being minimal. It is likely that the model captures the traits of batches A–E, which are similar, better than those of batch F. This raises the question, can the model and conclusions in this work be applied to other cultivars, and other batches? Recalibration is one of the leading approach to adapt old NIR models to new variations, wherein some approaches completely recalibrate the model parameters^74^, while other techniques offer parameter-free calibration enhancement^71,75^. Although not addressed in this work, this is a natural future direction.

Since lowering of water content amplifies NIR absorption features by other compounds, the NIR spectra of the susceptible sepals is likely a mixed response to multiple compounds. Also, the NIR range near 1400 nm is only one of the water absorption regions. Another region, which is captured by the current sensor, is near 1200 nm. However, model interpretation methods do not place significant relevance to this region. Therefore, although presence of water appears to be an indicator of a sepal’s health, it cannot be stated as a conclusion.

## Conclusions

This paper presents an automated approach to predict the susceptibility of the sepals of recently harvested tomatoes to future fungal infections. The proposed approach is a combination of NIR hyperspectral imaging (1000–1700 nm), data preprocessing pipeline and machine learning based modelling.

An experiment was designed where 6 batches of recently harvested cocktail tomatoes (cultivar Brioso), obtained from 5 growers based in The Netherlands and Belgium, were imaged using a hyperspectral camera before there was any evidence of visual fungal infection, leading to the hyperspectral dataset. These tomatoes were then placed for 4 days under controlled conditions with 100% humidity, an ideal setting for the fungal germination. After 4 days, almost all sepals were observed to have had fungal infection to a certain degree, and to objectively capture the degree of infection these tomatoes were imaged using a color camera. The severity of infection of each sepal was ranked by 11 annotators, and the final severity value for each sepal was fused using principal component analysis, by projecting the infection grade onto the first component.

A novel data pre-processing pipeline is proposed for automated segmentation the calyx and the individual sepals from the hyperspectral images. From data of each sepal two spectral features are computed, the mean spectra over the sepal and the standard deviation of the spectra across the sepal. Two ensemble machine learning methods, Random Forest and XGBoost, were investigated to model the relation between the spectral features and the fungal severity observed 4 days later. With the Random Forest regressor, a Pearson correlation of 0.82 is achieved, demonstrating a strong linear relationship between the spectral features and the eventual fungal severity. The sepal level results were averaged to predict the overall susceptibility of each tomato. A Pearson correlation of 0.92 was achieved, correlating the averaged human-annotated severity sepals from a tomato with the averaged prediction from the model. At the individual tomato as well as the batch level, the model predictions also had strong agreements with the ground truth. Such predictions, prior to the onset of infection, can offer key insights for post-harvest management of these tomatoes.

However, what does this correlation mean, and how can one interpret the results from this model? Another key contribution of the paper is the focus on model interpretation, with a focus on understanding which spectral features contribute the most to the model’s prediction. The contribution of individual spectral features in model decision was investigated using SHAP^63,64^ and Random Forest’s inbuilt feature importance metric. Using both approaches, a clear conclusion could be derived that not all wavelengths were relevant, and the NIR spectra between the 1390–1420 nm wavelength range contributed most to the model’s final decision. From an application perspective, this article highlights the importance of wavelengths ranging from 1390 to 1420 nm. A model trained on the features derived from this spectral region only slightly decreases in performance (Pearson correlation of 0.78) compared to the model based on the whole spectral range (Pearson correlation of 0.82).

From a chemometric perspective, NIR region identified in this paper corresponds mainly to one of the typical water absorption regions. However, another water absorption region, near 1200 nm, showed no significant contribution to model’s prediction. Therefore, it can be concluded that water content alone is not sufficient indicator of a sepal’s sensitivity to future fungal infection. From a practical view, identification of a limited set of wavelengths also has potential benefits. It is feasible to develop dedicated sensors, such as multi-spectral cameras, which include only the most relevant spectral wavelengths. This can lead to a practicable non-invasive sensor for mass inspections of recently harvested tomatoes for cultivar Brioso.

It must be stated that the contributions and conclusions drawn, correspond to the data acquired under this research, which relates to cocktail tomatoes from cultivar Brioso. How applicable are these results for other cultivars? This is the key inquiry not explored in this work, and provides a perspective for future research direction. There are two clear future directions: investigation of the suitability of identified NIR range to other tomato cultivars, and exploration of calibration transfer approaches to extend the current model to new cultivars.
